# The Choice of a Doctor's Motor-Car

**Published:** 1910-08-13

**Authors:** C. E. Price


### Motoring Notes.

### THE CHOICE OF A DOCTOR'S MOTOR-CAR.

By C. E. Price, M.R.C.S.Eng., L.R.C.P.Lond.

It is always a matter of interest to medical men who contemplate the acquisition of a car to learn the experiences of fellow medical men who already possess them.

The average doctor is so rarely of a mechanical bent of mind that the various considerations of make, style, and horse-power invariably confuse him, and the advice which he seeks from motor-users usually makes the confusion worse. Each adviser has his own opinion, and the variety of opinions throws the purchaser back upon his own powers of dissecting this mass of information.

### Personal Selection.

I will in this article give my experience of the purchase and use of my own car, after first stating that I was trained as a mechanical engineer before commencing my study in medicine. My practice is a rural one in Kent, and when I first decided to get a motor-car I sought advice from various friends who owned cars. They all warned me not to get a car of less than 15 h.p., as nothing under that power would enable me to surmount hills; also not to get a car with fewer than four cylinders, because all others were noisy. I consulted catalogues innumerable, and did the usual tour of the Olympia Show. The result was distraction of mind, and a desire for more and more power, although valuable information was gained by visiting the show. I did not make the mistake of ordering the car at the show, but resolved to wait, use my own discretion, and weigh the pros and cons of every make, keeping in mind my own requirements, and the cost of purchase and upkeep. It was necessary to have a four-seated car, as I wished, in addition to using it for professional purposes, to tour with my wife for our holidays, and to carry luggage. I wished also to have seating accommodation for friends if necessary. In the matter of the purchase of a car first cost ought not to weigh so much as the cost of upkeep, provided that a first-class car is obtained. The first cost and wear of tyres should receive careful consideration, and the greater the power of the car the greater is the wear and the less the life of the tyres, as well as the increased cost for renewal. The car had to be of such dimensions as to be convenient for medical work, to make reasonable speed on the road, and to be economical in wear and renewal of tyres.

Since I intended to be my own driver, and to attend the car myself, I decided that it would be far easier to look after one cylinder than four, one sparking plug than four, with all the electric-wire connections, and also to attend to two valves rather than eight.

### The Chosen Car.

I could not afford to try experiments, and so decided to get a car of known and tried quality and reliability, and my choice was the well-known De Dion Bouton, 8 h.p., single cylinder, 1910 model, which I purchased through Mr. A. E. Jones, of Bromley Common, the courteous Kentish agent for the London house. A trial car of similar type easily ran up Westerham Hill, a gradient of 1 in 7, with four passengers in the car.

The new car was delivered early in May of this year, in charge of one of the drivers of the firm, who remained for three days to give me some instruction with regard to driving, and so simple was the mechanism that I drove the car on the first day of delivery, and on the second day I drove my wife eighty miles through Kent.

The wisdom of the choice of this car has been demonstrated in its working. Since delivery it has run 1,500 miles at an average speed of eighteen miles an hour, including ten-mile limits, hills, and stoppages.

### The Question of Expense.

The original cost was £336 10s., including a four-seated side entrance body, with shields to front and back wings, extra finish to upholstery, Auster folding wind screen, Cape cart hood, and high doors to front seats. The tyres were larger than standard size, being Dunlop grooved, 760 mm. by 90 mm., with extra heavy treads. It was fitted with a Stepney wheel, and a horn and speedometer, and also included two acetylene headlights with generator, two oil side-lights and back light, and set of tools.

The running expenses up to the present have only involved the cost of petrol, and about eighteenpence a week for a boy to wash the car. The consumption of petrol has been 36 gallons to a mileage of 1,500 miles, which works out at 41.6 miles to the gallon, and I estimate that the annual cost for a mileage of 6,000 to 7,000 miles will not exceed £50, including tyre renewals, and insurance.

### Results.

The silence and smooth working of the car have surprised those who prophesied that the noise of a single-cylindered car would be unendurable. It has repeatedly been mistaken for a four-cylindered car, and its appearance with the side entrance body is such that it is constantly taken for a 15 h.p. car.

The tyres up to the present hardly show signs of wear beyond a few small cuts, and these I now deal with by using a vulcaniser for repair. The car can climb most hills on top speed, and any hill on second or first speed.

These results show the economy of using a small-power car, and such a car to the busy doctor is an invaluable aid in getting his work done at a reasonable hour, and the health, pleasure, and relaxation of mind that can be obtained in taking excursions and week-ends at the sea is just that little beneficial break in the monotony of practice that keeps the overworked practitioner fit and well in these days of stress.

